# Maintenance of human amnion epithelial cell phenotype in pulmonary surfactant

**DOI:** 10.1186/scrt495

**Published:** 2014-09-04

**Authors:** Courtney A McDonald, Jacqueline M Melville, Graeme R Polglase, Graham Jenkin, Timothy JM Moss

**Affiliations:** The Ritchie Centre, MIMR-PHI Institute of Medical Research, 27-31 Wright Street, Clayton, VIC 3168 Australia

## Abstract

**Introduction:**

Preterm newborns often require mechanical respiratory support that can result in ventilation-induced lung injury (VILI), despite exogenous surfactant treatment. Human amnion epithelial cells (hAECs) reduce lung inflammation and resultant abnormal lung development in preterm animals; co-administration with surfactant is a potential therapeutic strategy. We aimed to determine whether hAECs remain viable and maintain function after combination with surfactant.

**Methods:**

hAECs were incubated in surfactant (Curosurf) or phosphate-buffered saline (PBS) for 30 minutes at 37°C. Cell viability, phenotype (by flow cytometry), inhibition of T-cell proliferative responses and differentiation into lung epithelium-like cells (assessed with immunohistochemical staining of surfactant protein (SP)-A) were investigated.

**Results:**

Cell viability and apoptosis of hAECs were not altered by surfactant, and hAEC phenotype was not altered. hAECs maintained expression of epithelial cell adhesion molecule (EpCAM) and human leukocyte antigen (HLA)-ABC after surfactant exposure. Expression of HLA-DR, CD80 and CD86 was not increased. Immunosuppression of T cells by hAECs was not altered by surfactant. hAEC differentiation into lung epithelium-like cells was equivalent after exposure to PBS or surfactant, and SP-A expression was equivalent.

**Conclusion:**

Surfactant exposure does not alter viability or function of hAECs. Thus a combination therapy of hAECs and surfactant may be an efficacious therapy to ameliorate or prevent preterm lung disease.

## Introduction

Infants born preterm (before 37 completed weeks of gestation) lack pulmonary surfactant; a lipoprotein complex produced by type II alveolar epithelial cells [1] that reduces surface tension at the air-to-fluid interface. More than half of neonates born sooner than 28 weeks’ gestation require mechanical ventilation for sufficient gas exchange [2], which can cause ventilation-induced lung injury (VILI) and contribute to life-threatening bronchopulmonary dysplasia (BPD). Administration of exogenous surfactant (purified from animals and composed of phospholipids and some of the proteins contained in the original material) to preterm infants improves their respiratory status but does not prevent BPD [3]. Treatment of ventilated lambs with exogenous surfactant reduces inflammation [4], but it does not prevent VILI [3].

Cell therapy presents an attractive option for treatment of VILI and BPD. We recently demonstrated the capacity of human amnion epithelial cells (hAECs) to prevent VILI and the BPD phenotype in experimental animal models [5–8].

hAECs compose the inner surface of the amnion [9, 10], and are able to differentiate into cell types of all three germ layers [11, 12], including neuronal cells, smooth muscle, cardiomyocytes, osteocytes, adipocytes, hepatocytes, and pancreatic cells [10, 11]. hAECs can be induced to differentiate into lung epithelium-like cells *in vitro* [13]. Unlike other pluripotent cells, such as embryonic stem cells, hAECs do not form teratomas [10].

Exogenous surfactant is a potential vehicle for the administration of hAECs to preterm neonates. Therefore, we aimed to determine whether surfactant exposure, at a concentration administered clinically, altered hAEC viability, phenotype, and function. We hypothesized that surfactant exposure would not be detrimental to hAECs, thereby demonstrating the potential for a novel combination therapy for VILI.

## Methods

### Isolation of hAECs and surfactant treatment

All experiments were performed with approval from the Monash University Human Ethics Committee. Human AECs were isolated as previously described [14]. In brief, placentae were obtained from women with uncomplicated pregnancies undergoing elective caesarean section at term. Women gave written, informed consent for the collection of their placentae. The amnion was manually stripped from the chorion, and the hAECs enzymatically removed from the amnion by two, 1-hour digestions in Trypzean (Sigma-Aldrich, Sydney, Australia). Trypzean was inactivated by Soybean trypsin inhibitor (Sigma-Aldrich), and the hAECs collected by centrifugation. Live-cell counts and viability were determined by trypan blue exclusion. For cryopreservation, hAECs were frozen at a density of 5 × 10^6^ cells/ml; media consisted of 90% fetal bovine serum (FBS; Gibco, Life Technologies) and 10% dimethyl sulfoxide (DMSO; Sigma Aldrich). Cells were then transferred to freezer tubes and left in a freezing container (MrFrosty, Thermo Fisher Scientific) overnight at -80°C, after which they were transferred to liquid nitrogen. To thaw, hAEC sample tubes were quickly removed from liquid nitrogen and placed directly into a 37°C water-bath until thawed. Samples were washed to remove DMSO, and cell counts and viability were determined. Approximately 15 × 10^6^ hAECs in 1 ml phosphate-buffered saline (PBS) were exposed to either 1 ml of surfactant (Curosurf, kindly provided by Chiesi Pharmaceuticals, Italy) or PBS for 30 minutes in a 37°C water-bath. We considered that this period of incubation would be realistic in a clinical setting if hAECs were to be administered in surfactant.

After incubation, cells were washed with PBS and viability was again determined by using trypan blue exclusion.

### Flow cytometry

Phenotypic analysis was performed on hAECs after surfactant or PBS exposure alone. Single-color flow cytometry was performed by staining 5 × 10^5^ cells with primary antibody for 20 minutes at 4°C. The relevant isotype control antibody was used as a negative control. Cells were then washed with FACS buffer (1% FBS in PBS) and underwent centrifugation at 300 rcf for 5 minutes at 4°C. Data acquisition was performed by using a FACSCanto II flow cytometer, and data were analyzed by using Flowlogic Software (Inivai Technologies, Mentone, VIC, Australia). All primary antibodies were purchased from BD Biosciences, Australia.

### Proliferation assays

Proliferation assays were performed as previously described [15]. In brief, splenocytes were isolated from C57BL/6 adult mice or fetal sheep and seeded in 96-well, flat-bottom microtiter plates (Nunc, Thermo Fisher Scientific, Australia). Assays were performed in triplicate at a concentration of 2.5 × 10^5^ cells per well in complete RPMI medium alone or in the presence of 5 μl/ml concanavalin A (ConA; Sigma-Aldrich), or into wells precoated with 10 μg/ml anti-CD3 (clone 145-2C11) and 10 μg/ml anti-CD28 (clone 37.51) antibodies (both from BD Biosciences) to a final volume of 200 μl per well. For wells that required addition of hAECs, 50 μl of hAECs that had been exposed to surfactant or PBS, at hAEC-to-splenocyte ratios ranging from 1:5 to 1:40, were added to each well before the addition of splenocytes. Cells were incubated at 37°C for 48 hours and then 1 μCi/well [^3^H]-thymidine (Perkin Elmer, Waltham, MA, USA) was added for an additional 18 hours of culture. Cells were harvested onto filter mats (Perkin Elmer), and incorporated radioactive nucleic acids were counted by using a Top Count Harvester (Packard Biosciences, Meriden, CT, USA).

### Wound-healing assay

A scratch assay was used to assess the wound-healing properties of hAECs. After surfactant or PBS treatment, hAECs were plated into six-well plates at a density of 20,000 cells/cm^2^ and cultured in standard DMEM/F12 medium supplemented with 10% FBS (Gibco, Life Technologies). Cells were left until they became 100% confluent (approximately 10 to 12 days), and then a cross was scratched in the middle of the well by using a 1,000-μl pipette tip. Images were taken by using a phase-contrast microscope at the corner of the cross, so the exact position could be replicated. Cells were assessed and images were captured at 0 and 72 hours by using an Olympus CKZ41 inverted microscope (Olympus): the scratched area was quantified by using ImageJ (NIH [16]).

### Differentiation of hAECs to alveolar type II cells

After surfactant or PBS treatment, hAECs were plated into six-well plates containing 22-mm glass coverslips (Menzel-Glaser, Germany), at a density of 20,000 cells/cm^2^. hAECs were cultured in either Small Airway Epithelial Growth Medium (SAGM; Lonza Australia Pty Ltd, Australia) or DMEM/F12 with 10% FBS for up to 28 days without passage. For Surfactant Protein A (SP-A) immunostaining, hAECs were fixed in 4% paraformaldehyde in PBS for 15 minutes and permeabilized with 0.1% Triton-X-100 in PBS for 5 minutes at room temperature. DAKO Protein Block Serum-Free was used to block nonspecific binding (10 minutes at room temperature).

Cells were incubated overnight at 4°C in anti-SP-A antibody (Millipore). Alexa Fluor 488 goat anti-mouse IgG (Life Technologies) was used as a secondary antibody and was incubated for 1 hour at room temperature. Cells were counterstained with Hoechst (Invitrogen, Australia) for 10 minutes at room temperature. Imaging was performed by using a Nikon C1 laser scanning microscope.

### Statistical analysis

Results are expressed as mean ± standard error of the mean (SEM). Statistical analysis was performed with Prism 5.03 (GraphPad Software). Experimental and control groups were compared with paired or unpaired *t* test, or one-way ANOVA (with Bonferroni *post hoc* analysis), where appropriate. A value of *P* < 0.05 was considered statistically significant.

## Results

### Cell viability after surfactant treatment

**Figure 1:**
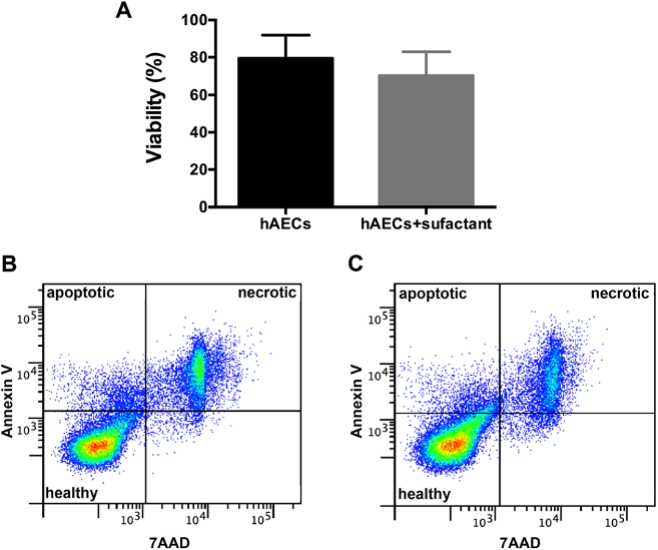
**Surfactant treatment does not reduce hAEC viability. (A)** Cell viability of hAECs incubated with either PBS or surfactant (*n* = 6). **(B)** Representative flow-cytometry plot assessing proportions of apoptotic and necrotic hAECs after PBS exposure. **(C)** Representative flow-cytometry plot assessing proportions of apoptotic and necrotic hAECs after surfactant exposure.

No difference was observed in hAEC viability (from six donors) after either surfactant or PBS exposure (Figure 1A). The proportions of apoptotic or necrotic cells were not different between hAECs exposed to PBS (Figure 1B) or to surfactant (Figure 1C).

### Expression of hAEC cell-surface markers after surfactant treatment

Expression of epithelial cell adhesion molecule (EpCAM) was high on hAECs (isolated from three donors) and not reduced by surfactant (72% ± 4% and 70% ± 5% for PBS- and surfactant-exposed, respectively). All hAEC samples had high levels (>50%) of human leukocyte antigen (HLA)-ABC and, after surfactant exposure, cells from two of the three donors reduced expression to less than 15% (expression in the other donor was unchanged). hAECs were negative for HLA-DR and co-stimulatory markers CD80 and CD86, and had negligible expression of CD90, CD44, and CXCR4, regardless of surfactant exposure (Table 1).

**Table 1:** **Expression of hAEC surface markers after surfactant treatment**

Surface marker	hAEC			hAEC + surfactant		
	i	ii	iii	i	ii	iii
EpCAM	++	++	++	++	++	++
HLA-ABC	++	++	++	+	++	+
HLA-DR	-	-	-	-	-	-
CD80	-	-	-	-	-	-
CD86	-	-	-	-	-	-
CD90	-	-	-	+	-	-
CD44	-	-	-	-	-	+
CD184 (CXCR4)	+	-	-	-	-	-

Cell-surface expression as assessed with flow cytometry. –, not detected; +, low (5% to 15%); ++, high (>50%).

### Effect of surfactant treatment on immunosuppressive properties of hAECs

**Figure 2:**
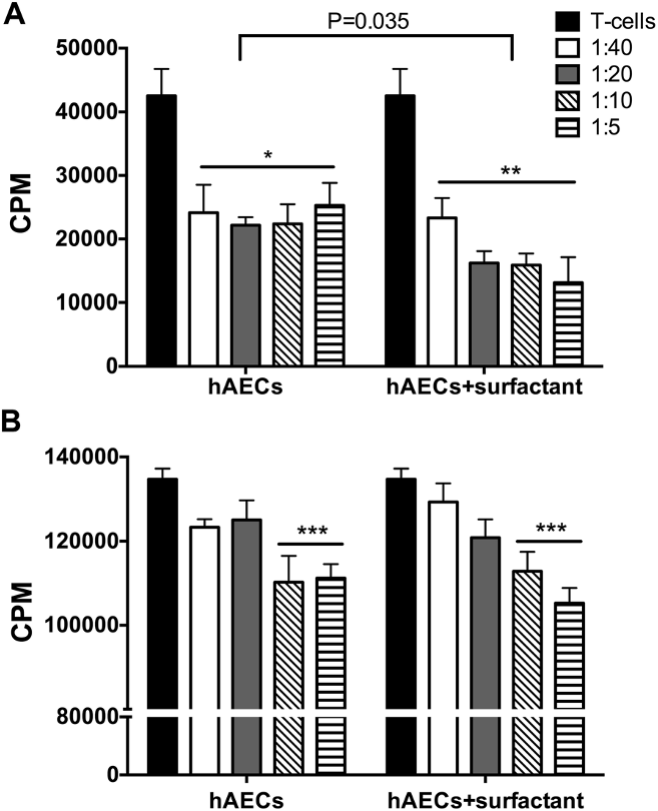
**hAECs retain immunosuppressive properties after surfactant treatment. (A)** Sheep T cells were stimulated with Con A alone or after coculture with hAECs after treatment with PBS or surfactant (*n* = 9, performed in triplicate; *P < 0.05 **P < 0.01). **(B)** Mouse T cells were stimulated with anti-CD3/anti-CD alone or after coculture with hAECs after treatment with PBS or surfactant (*n* = 9, performed in triplicate. ***P < 0.001.

Sheep splenocyte proliferation in response to Con A (Figure 2A) was significantly reduced by hAECs (*P* < 0.05, *P* < 0.01, respectively), but inhibition was greater for hAECs exposed to surfactant (*P* < 0.035). Mouse splenocyte proliferative responses were significantly reduced by hAECs alone or by surfactant-exposed hAECs (Figure 2B; *P* < 0.001).

### Effect of surfactant treatment on the wound-healing properties of hAECs

**Figure 3:**
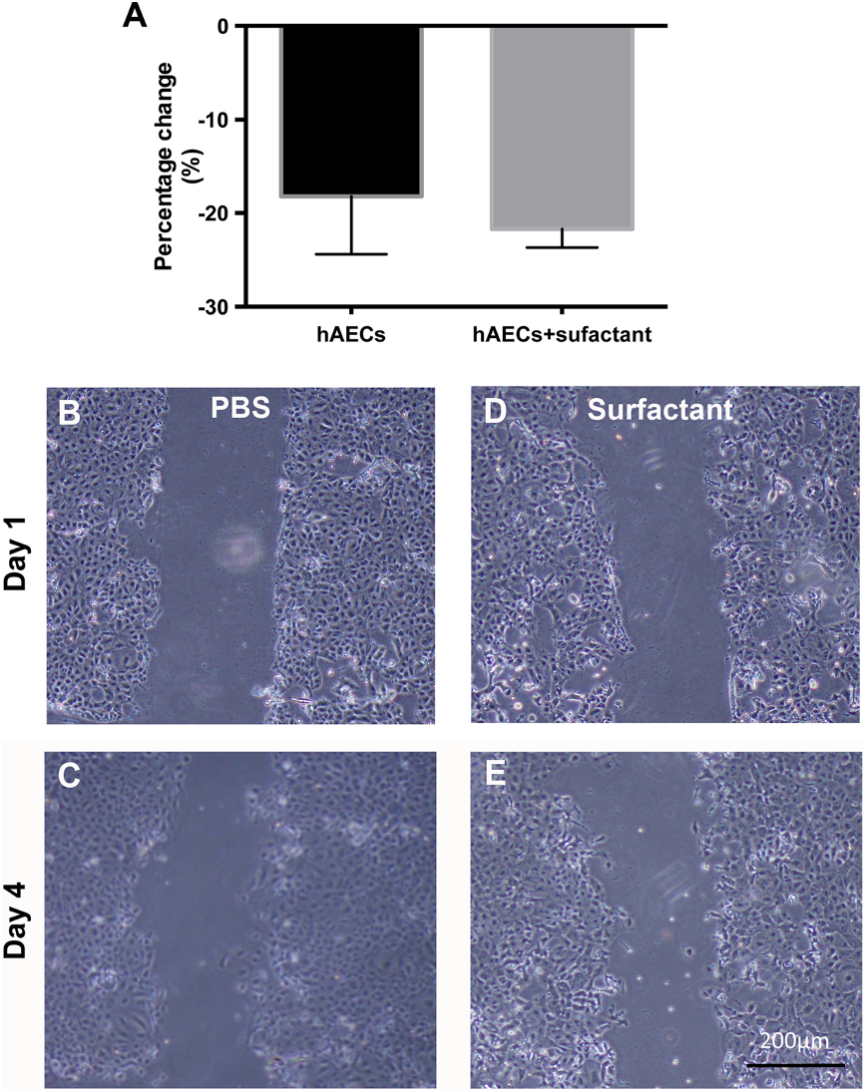
**Surfactant treatment does not affect wound healing and migration.** Cellular migration was measured by using a scratch-wound assay with hAECs treated with PBS or surfactant. **(A)** Percentage change in the wound area for hAECs exposed to PBS or surfactant (n = 3). **(B)** Representative image of PBS-treated hAECs at 0 hours and **(C)** 72 hours. **(D)** Representative image of surfactant-treated hAECs at 0 hours and **(E)** 72 hours.

Wound area was reduced 18.2% ± 3.6% and 21.7% ± 1.1% for hAECs exposed to PBS or surfactant, respectively (Figure 3A): this difference was not statistically significant.

### Effect of surfactant treatment on the alveolar cell differentiation potential of hAECs

**Figure 4:**
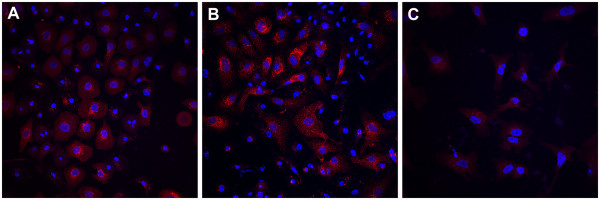
**Surfactant-treated hAECs retain the ability to differentiate into type II alveolar cells. (A)** PBS-treated hAECs cultured in SAGM for 28 days. **(B)** Surfactant-treated hAECs cultured in SAGM for 28 days. **(C)** hAECs cultured in control media (DMEM/F12) for 28 days. Red stain denotes expression of surfactant protein-A, and Hoechst (blue) staining was used to label cell nucleus (*n* = 3, performed in duplicate for all conditions).

hAECs exposed to either PBS (Figure 4A) or surfactant (Figure 4B) readily differentiated to type II alveolar epithelium-like cells, as demonstrated by SP-A expression. hAECs cultured in control medium did not express SP-A, regardless of treatment (Figure 4C).

## Discussion

Cell therapy is rapidly nearing translation into treatment to improve lung development and prevent the progression to BPD in preterm infants who require invasive respiratory support. Combination therapy with prophylactic surfactant is a logical combination. We have shown that exposure of hAECs to surfactant has no detrimental impact on cell viability, phenotype, or function. Moreover, hAECs retained their ability to suppress nonspecific and T-cell receptor-specific immune responses, and surfactant-exposed hAECs were able to differentiate into type II-like alveolar epithelial cells *in vitro*.

Inflammation and immune cell activation appear critical for the development of lung injury during mechanical ventilation, which induces epithelial cell damage, protein leak, and neutrophil migration into the lung, as well as elevated gene expression of inflammatory cytokines interleukin (IL)-1β, IL-6, and serum amyloid A3 in lung tissue [17–19]. Animal studies have shown that cell therapy with hAECs decreased lung injury and inflammation in hyperoxia-exposed mice [20] and in a bleomycin model of fibrotic lung injury [7]. We have shown that hAECs exposed to surfactant, at concentrations used clinically, does not reduce the ability of the cells to suppress T-cell immune responses and therefore would be unlikely to reduce their *in vivo* immunomodulation capacity.

Immunomodulation is an important aspect of the action of hAECs [21, 22]. hAECs are able to modulate lung inflammation through polarization of macrophages toward a reparative M2 phenotype [23]. Furthermore, hAECs reduce proliferation and the production of inflammatory cytokines in T-cell cocultures [24, 25], and our studies have shown that hAECs exposed to surfactant still retain the ability to suppress T-cell responses.

Another important property of hAECs is their ability to differentiate into type II alveolar cells *in vitro* [14] and *in vivo* [6, 8]. Type II alveolar cells are the sites of production, secretion, and recycling of surfactant [1, 26]. The preterm lungs do not produce sufficient surfactant to maintain lung integrity; thus the ability of exogenously administered cells to differentiate into functional surfactant-producing cells may be beneficial. In ventilated preterm lambs, carboxyfluorescein succinimidyl ester (CFSE)-labeled hAECs administered intratracheally engrafted in small numbers and differentiated into alveolar type II cells, as evidenced by the expression of pro-surfactant-C [8]. Our study demonstrates that hAECs can differentiate into type II alveolar epithelial cells and that exposure to surfactant does not inhibit the ability of hAECs to undergo differentiation.

Although engraftment and differentiation of hAECs may occur *in vivo,* it has recently become evident that significant cell engraftment does not normally occur in experimental lung injury [27]. It is more likely that hAECs act through paracrine effects and may also recruit endogenous stem cells to replace damaged tissue [28]. Recently, by using a hyperoxic rodent model of BPD, it was shown that mesenchymal stem cells (MSCs) were able to reduce lung injury, in part, through activation and recruitment of endogenous bronchoalveolar stem cells to the site of injury [29]. Although this phenomenon in the lung has not been investigated after hAEC administration, hAECs can promote host repair in a monkey model of spinal cord injury [30], potentially through the recruitment of neural precursor cells.

hAECs are generally classified on the basis of their epithelium-like morphology and expression of EpCAM [14]. In this study, we verified that hAECs exposed to surfactant retained their epithelial morphology and the expression of EpCAM. hAECs are generally regarded as immunoprivileged cells that are negative for HLA class II and co-stimulatory molecules, and have limited alloimmune responses. Flow-cytometric analysis revealed that hAECs exposed to surfactant did not upregulate any co-stimulatory markers (HLA-DR, CD80, CD86), suggesting that they retain their immunoprivileged properties.

Allografts of AECs in mice corneas produced delayed hypersensitivity to donor tissue 2 weeks after engraftment, but not after 4 or 8 weeks [31]. This implies that memory responses against the allograft are not produced despite initial sensitization. It has been proposed that this lack of hypersensitivity to allografts of hAECs is due to lack of HLA class II and co-stimulatory molecule expression and limited expression of HLA class I [32]. Unlike other studies, our results suggest that untreated hAECs indeed express HLA class I antigens, and this was not further upregulated by surfactant exposure.

We also investigated expression of CD90, CD44, and CD184. CD90 and CD44 are predominantly used as markers of MSCs; however, it has been shown that CD90 is upregulated on hAECs after prolonged time in culture [33]. We found hAECs were negative for both CD90 and CD44, and expression was not altered by surfactant exposure. CD184 (also known as CXCR4) is an important chemokine receptor that can be upregulated on MSCs [34] and plays a role in the homing of cells to areas of brain injury [35, 36]. The expression of CD184 has not been studied on hAECs previously; our results suggest that hAECs do not express CD184 on their surface and do not upregulate this receptor after surfactant exposure.

## Conclusion

Human amnion epithelial cells (hAECs) are viable, and their function is preserved, in pulmonary surfactant. Administration of hAECs in exogenous surfactant is a realistic treatment strategy for neonatal respiratory disease.

## Authors’ information

Courtney A McDonald and Jacqueline M Melville are Joint first authors.

## Abbreviations

BPD: Bronchopulmonary dysplasia
CFSE: carboxyfluorescein succinimidyl ester
hAEC: human amnion epithelial cell
HLA: human leukocyte antigen
IL: interleukin
MSC: mesenchymal stem cell
SP: surfactant protein
VILI: ventilation-induced lung injury.
